# Diverse spectrum of rare deafness genes underlies early-childhood hearing loss in Japanese patients: a cross-sectional, multi-center next-generation sequencing study

**DOI:** 10.1186/1750-1172-8-172

**Published:** 2013-10-28

**Authors:** Hideki Mutai, Naohiro Suzuki, Atsushi Shimizu, Chiharu Torii, Kazunori Namba, Noriko Morimoto, Jun Kudoh, Kimitaka Kaga, Kenjiro Kosaki, Tatsuo Matsunaga

**Affiliations:** 1 Laboratory of Auditory Disorders, National Institute of Sensory Organs, National Hospital Organization Tokyo Medical Center, 2-5-1 Higashigaoka, Meguro, Tokyo 152-8902, Japan; 2 Iwate Tohoku Medical Megabank Organization, Iwate Medical University, Iwate, Japan; 3 Center for Medical Genetics Keio University School of Medicine, Tokyo, Japan; 4 Department of Otorhinolaryngology, National Center for Child Health and Development, Tokyo, Japan; 5 Laboratory of Gene Medicine, Keio University School of Medicine, Tokyo, Japan; 6 National Institute of Sensory Organs, National Hospital Organization Tokyo Medical Center, Tokyo, Japan

**Keywords:** Hereditary hearing loss, Target gene capture, Deafness gene, Heterogeneity

## Abstract

**Background:**

Genetic tests for hereditary hearing loss inform clinical management of patients and can provide the first step in the development of therapeutics. However, comprehensive genetic tests for deafness genes by Sanger sequencing is extremely expensive and time-consuming. Next-generation sequencing (NGS) technology is advantageous for genetic diagnosis of heterogeneous diseases that involve numerous causative genes.

**Methods:**

Genomic DNA samples from 58 subjects with hearing loss from 15 unrelated Japanese families were subjected to NGS to identify the genetic causes of hearing loss. Subjects did not have pathogenic *GJB2* mutations (the gene most often associated with inherited hearing loss), mitochondrial m.1555A>G or 3243A>G mutations, enlarged vestibular aqueduct, or auditory neuropathy. Clinical features of subjects were obtained from medical records. Genomic DNA was subjected to a custom-designed SureSelect Target Enrichment System to capture coding exons and proximal flanking intronic sequences of 84 genes responsible for nonsyndromic or syndromic hearing loss, and DNA was sequenced by Illumina GAIIx (paired-end read). The sequences were mapped and quality-checked using the programs BWA, Novoalign, Picard, and GATK, and analyzed by Avadis NGS.

**Results:**

Candidate genes were identified in 7 of the 15 families. These genes were *ACTG1, DFNA5, POU4F3*, *SLC26A5*, *SIX1, MYO7A*, *CDH23*, *PCDH15*, and *USH2A*, suggesting that a variety of genes underlie early-childhood hearing loss in Japanese patients. Mutations in Usher syndrome-related genes were detected in three families, including one double heterozygous mutation of *CDH23* and *PCDH15*.

**Conclusion:**

Targeted NGS analysis revealed a diverse spectrum of rare deafness genes in Japanese subjects and underscores implications for efficient genetic testing.

## Background

Hearing loss is a common sensory defect, affecting approximately one in 500 to 1000 newborns [1]. Approximately 50% of congenital hearing loss cases and 70% of childhood hearing loss cases are attributed to genetic mutations [1]. The remaining 50% of congenital cases are attributable to other factors such as prenatal exposure to measles, cytomegalovirus, premature birth, and newborn meningitis. Genetic tests for hereditary hearing loss assist in the clinical management of patients and can provide the first step in the development of therapeutics [2]. For example, early diagnosis of Usher syndrome, which comprises congenital hearing loss and late-onset retinitis pigmentosa, provides important information to choose communication modalities. However, causes of hereditary hearing loss are highly heterogeneous; more than 60 genes have been identified as responsible for nonsyndromic hearing loss [3], and several hundreds of syndromic diseases, such as Pendred syndrome and Usher syndrome, are accompanied by hearing loss. *GJB2* mutations are the most common cause of childhood hearing loss worldwide [1], followed by *SLC26A4* mutations [4]. *OTOF* mutations are common in patients with auditory neuropathy, which is characterized by normal outer hair cell function and abnormal neural conduction [5]. The prevalence of childhood hearing loss patients with mutations in other deafness-related genes is likely to be less than 1% [1]. Such high heterogeneity of hearing loss makes it impractical to perform genetic tests by Sanger sequencing. This is also the case for some types of syndromic hearing loss. For example, nine genes have been reported to cause Usher syndrome, and all are large and difficult to analyze using Sanger sequencing.

Next-generation sequencing (NGS) technology has been applied to genetic diagnosis of nonsyndromic hearing loss [6-8] and exploring the causes of hearing loss [9-11]. These studies have revealed that it is technically feasible to identify causative genes for nonsyndromic and syndromic hearing loss using targeted NGS [6,8]. In this study, we used targeted NGS to identify the genetic basis of hearing loss in Japanese families.

## Methods

### Subjects

This was a multi-center study of 58 subjects (36 subjects with hearing loss and 22 subjects with normal hearing) from 15 unrelated Japanese families in which at least two family members had bilateral hearing loss. All subjects were patients at the National Hospital Organization Tokyo Medical Center or a collaborating hospital. Medical histories were obtained and physical, audiological, and radiological examinations were carried out for the subjects and family members. Subjects with hearing loss related to environmental factors were excluded. Subjects with *GJB2* mutations or mitochondrial m.1555A>G or 3243A>G mutations were excluded. Subjects with enlarged vestibular aqueduct, which is often associated with *SLC26A4* mutations, and subjects with clinical features that suggested syndromic hearing loss were excluded. Subjects with auditory neuropathy were tested for *OTOF* mutations, which are associated with auditory neuropathy [12], and subjects with *OTOF* mutations were excluded. The Ethics Review Committees of the National Hospital Organization Tokyo Medical Center and all collaborating hospitals approved the study procedures. All procedures were conducted after written informed consent had been obtained from each subject or their parents.

### Targeted capture and DNA sequencing

We selected coding exons and proximal flanking intronic sequences of 84 genes, including 17 genes responsible for autosomal dominant nonsyndromic hearing loss (DFNA), 32 genes responsible for autosomal recessive nonsyndromic hearing loss (DFNB), 8 genes responsible for both DFNA and DFNB, one gene responsible for auditory neuropathy, 3 genes responsible for X-linked hearing loss, and 23 genes responsible for syndromic hearing loss. A list of the targeted genes responsible for nonsyndromic or syndromic hearing loss is provided in the supporting material [Additional file 1]. More than 90% of the target genomic sequences were successfully designed to be captured by the SureSelect Target Enrichment System (Agilent Technologies, CA, USA) (data not shown). Genomic DNA was extracted from whole blood using the Genetra Puregene DNA isolation kit (QIAGEN, Hilden, Germany) and checked for quality using Qubit (Life technologies, CA, USA). Genomic DNA (3 μg) was fragmented into approximately 150 base pairs and used to capture the targeted genomic sequences. The captured DNA was subjected to the paired-end read sequencing system (GAIIx system; Illumina, CA, USA).

### Sequence analysis

Sequence analysis initially focused on the 61 genes responsible for nonsyndromic hearing loss. If no candidate mutations were detected among these genes, the 23 genes responsible for syndromic hearing loss were subjected to sequence analysis.

The sequences were mapped and quality-checked with the programs BWA, Novoalign, Picard, and GATK using the human reference sequence hg19/GRCh37. Single and multiple nucleotide variants, including small insertion or deletions that would affect amino acid sequences or could affect splice sites, were annotated by Avadis NGS v.1.4.5 (Strand Life Sciences, Bangalore, India). Variants already known as pathogenic mutations or detected with <1% frequency in public databases (dbSNP135 [13], 1000GENOME [14], NHLBI Exome Variant Server [15]) were extracted and further subjected to segregation analysis within each family. If no candidate variants were found, the 23 genes responsible for syndromic hearing loss were subjected to the same procedures.

Selected variants were classified as known mutations, possible pathogenic mutations, or variants with unknown pathogenicity; the latter classification was made if there were reports of a controversial finding of pathogenicity or >1% allele frequency in the in-house database of 95 (up to 189) Japanese subjects with normal hearing. Conservation of the corresponding mutated amino acid was compared across nine primate, 20 mammal, and 13 vertebrate species by UCSC Conservation [16]. Functional pathogenic effects of the variants were predicted by PolyPhen-2 [17] and PROVEAN [18]. Effect on splice-site mutations was predicted by NNSPLICE [19].

All the variants and their segregation in each family were confirmed by Sanger sequencing. The specific primer sets were selected from the resequencing amplicon probe sets (NCBI) or designed originally by Primer-BLAST (NCBI). The genotype of each individual and segregation in the family was characterized using DNASIS Pro (Hitachisoft, Tokyo, Japan).

### Structural modeling

To find sequences homologous to ACTG1 and MYO7A that could be used as the structural templates for the modeling exercise, we searched the Protein Data Bank (PDB) using Gapped BLAST [20] and PDBsum [21]. The crystal structure of *Limulus polyphemus* filamentous actin (PDB: 3B63) and the 4.1 protein-ezrin-radixin-moesin (FERM) domain of *Mus musculus* myosin VIIa in complex with Sans protein (PDB: 3PVL) were utilized as the templates to model ACTG1 with the p.G268S mutation and MYO7A with the p.W2160G mutation, respectively. The models were built using SWISS-MODEL [22-24] in the automatic modeling mode and with default parameters. The quality of the models was evaluated using the Verify_3D Structure Evaluation Server [25,26]. The α-carbon frames and ribbon models were superimposed using Chimera [27].

## Results

**Figure 1:**
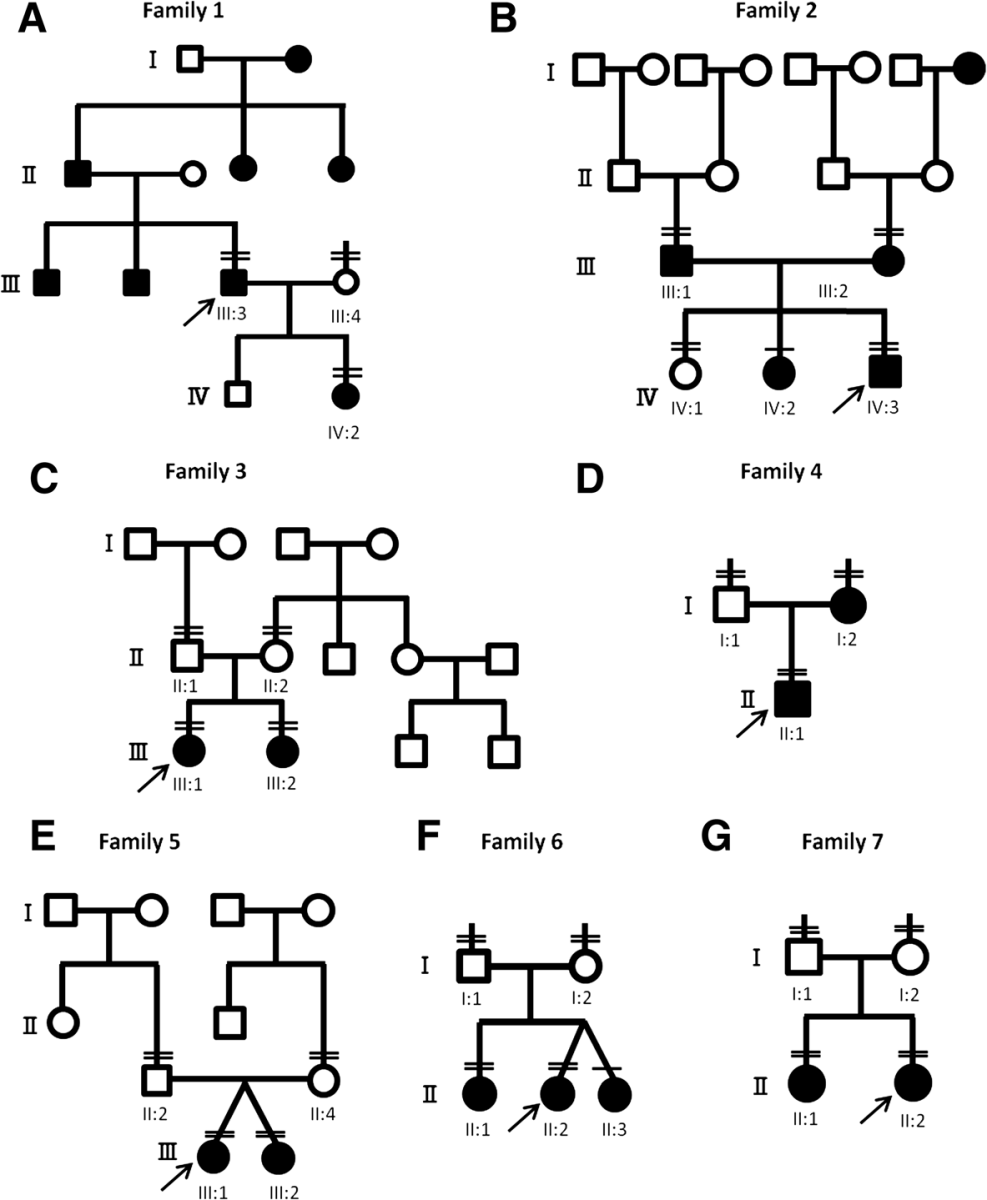
**Pedigrees of the seven families with hearing loss.** Double horizontal bars above a symbol indicate individuals who underwent genetic analysis by targeted next-generation sequencing. Single horizontal bars above a symbol indicate individuals who underwent analysis by Sanger sequencing. **A**-**G** denote pedigrees of family 1-7, respectively.

Pedigrees of the seven families are shown in Figure 1; clinical features are described in Table 1 and supplemental materials [Additional file 2 and Additional file 3]. In this targeted NGS study, the mean read depth of the target regions was more than 100× for all subjects (data not shown). Table 2 summarizes the number of variants detected from the 61 or 84 targeted genes for each subject. The number of variants was consistent across subjects (339–435 variants per subject for 61 genes, 539–607 variants per subject for 84 genes), which supported the reproducibility and reliability of our technical procedures and analytical pipeline. After excluding frequent variants (>1%) in public databases, 12 variants of 9 genes co-segregated with symptoms and were selected as possible pathogenic mutations (Table 3) or variants with uncertain pathogenicity in 7 families (Table 4).

**Table 1:** Summary of subjects with hearing loss

**Family**	**Subject**	**Age at onset (years)**	**Age at the time of the study (years)**	Hearing loss severity (left/right)*	**Progression**
1	III:3	45	53	Moderate/Moderate	Yes
	IV:2	10	16	Mild/Normal	No
2	III:1	unknown	no data	Profound/Profound	Unknown
	III:2	unknown	no data	Moderate/Severe	Unknown
	IV:3	0	1	Severe**	Yes
3	III:1	0	9	Severe**	Unknown
	III:2	0	6	Moderate/Moderate	Unknown
4	I:2	0	30s	Profound/Profound	No
	II:1	0	2	Profound/Profound	No
5	III:1	0	2	Severe**	No
	III:2	0	2	Profound**	No
6	II:1	5	14	Profound/Severe	Yes
	II:2	0	12	Profound/Profound	Yes
7	II:1	0	3	Moderate (ASSR***)	Unknown
	II:2	0	0	Severe (ASSR)	Unknown

*Hearing loss severity was evaluated based on average hearing level at 500, 1,000, 2,000, and 4,000 Hz (mild, 20–40 dB; moderate, 41–70 dB; severe, 71-95dB; profound, >95 dB) according to recommendations [3]. **Binaural hearing level. ***ASSR, auditory steady state responses.

**Table 2:** Summary of the number of variants detected in each subject

**Family**	**Subject**	**Number of genes analyzed**	No.SNV/MNV*	**No. non-synonymous SNV/MNV**
1	III:3	61	414	84
	III:4	61	370	74
	IV:2	61	391	82
2	III:1	61	386	81
	III:2	61	422	87
	IV:1	61	435	82
	IV:3	61	400	84
3	II:1	61	383	82
	II:2	61	339	70
	III:1	61	350	74
	III:2	61	398	86
4	I:1	84	570	138
	I:2	84	569	126
	II:1	84	546	131
5	II:2	61	388	72
	II:4	61	374	87
	III:1	61	361	84
	III:2	61	396	85
6	I:1	61	429	96
	I:2	61	371	81
	II:1	61	378	86
	II:2	61	375	84
7	I:1	84	607	139
	I:2	84	554	126
	II:2	84	582	132
	II:1	84	539	117

*SNV, single nucleotide variant; *MNV*, multiple nucleotide variant.

**Table 3:** Summary of possible pathogenic mutations

**Gene**	**Nucleotide change**	**Amino acid change**	**NCBI ID**	**dbSNP135**	**Allele frequency in 1000GENOME**	**Allele frequency in ESP6500**	**Allele frequency in Japanese control**	**PolyPhen-2 prediction (score)**	**PROVEAN prediction (score)**	**Pathogenicity**	**Family**	**Reference**
*ACTG1*	c.802G>A	p.G268S	NM_001199954.1	None	-	0	0/192	Probably damaging (0.998	Deleterious (-4.504)	Possible	1	
*POU4F3*	c.1007delC	p.A336Vfs	NM_002700.2	None	-	0	0/192	-	-	Possible	2	
*SLC26A5*	c.390A>C	p.R130S	NM_198999.2	None	-	0	0/192	Benign (0.443)	Deleterious (-4.813)	Possible	3	
*SLC26A5*	c.209G>A	p.W70X	NM_198999.2	None	-	0	n.t*.	-	-	Possible	3	
*SIX1*	c.328C>T	p.R110W	NM_005982.3	rs80356459	No info	0	n.t.	Probably damaging (1.000)	Deleterious (-7.775)	Causative	4	35
*MYO7A*	c.6478T>G	p.W2160G	NM_000260.3	None	-	0	0/192	Probably damaging (1.000)	Deleterious (-12.649)	Possible	5	
*MYO7A*	c.6439-2A>G (intron 51)	Splice mutation	NM_000260.3	None	-	0	0/192		-	Possible	5	
*CDH23*	c.719C>T	p.P240L	NM_022124.5	rs121908354	1/2183	0	n.t.	Probably damaging (1.000)	Deleterious (-3.051)	Causative	6	43
*PCDH15*	c.848G>A	p.R283H	NM_001142763.1	None	-	1/13005	0/192	Probably damaging (0.998)	Neutral (-1.918)	Possible	6	
*USH2A*	c.12431delC	p.A4144GfsX23	NM_206933.2	None	-	0	0/190		-	Possible	7	

*n.t. = not tested

**Table 4:** Summary of variants with uncertain pathogenicity

**Gene**	**Nucleotide change**	**Amino acid change**	**NCBI ID**	**dbSNP135**	**Allele frequency in 1000GENOME**	**Allele frequency in ESP6500**	**Allele frequency in Japanese control**	**PolyPhen-2 prediction (score)**	**PROVEAN prediction (score)**	**Pathogenicity**	**Family**	**Reference**
*DFNA5*	c.781C>T	p.R261X	NM_004403.2	None	-	0	0/192	-	-	Uncertain	2	
*USH2A*	c.1346G>A	p.R449H	NM_206933.2	None	-	0	5/378	Benign (0.017)	Neutral (-0.880)	Uncertain	7	

### Candidate mutations in each family

**Figure 2:**
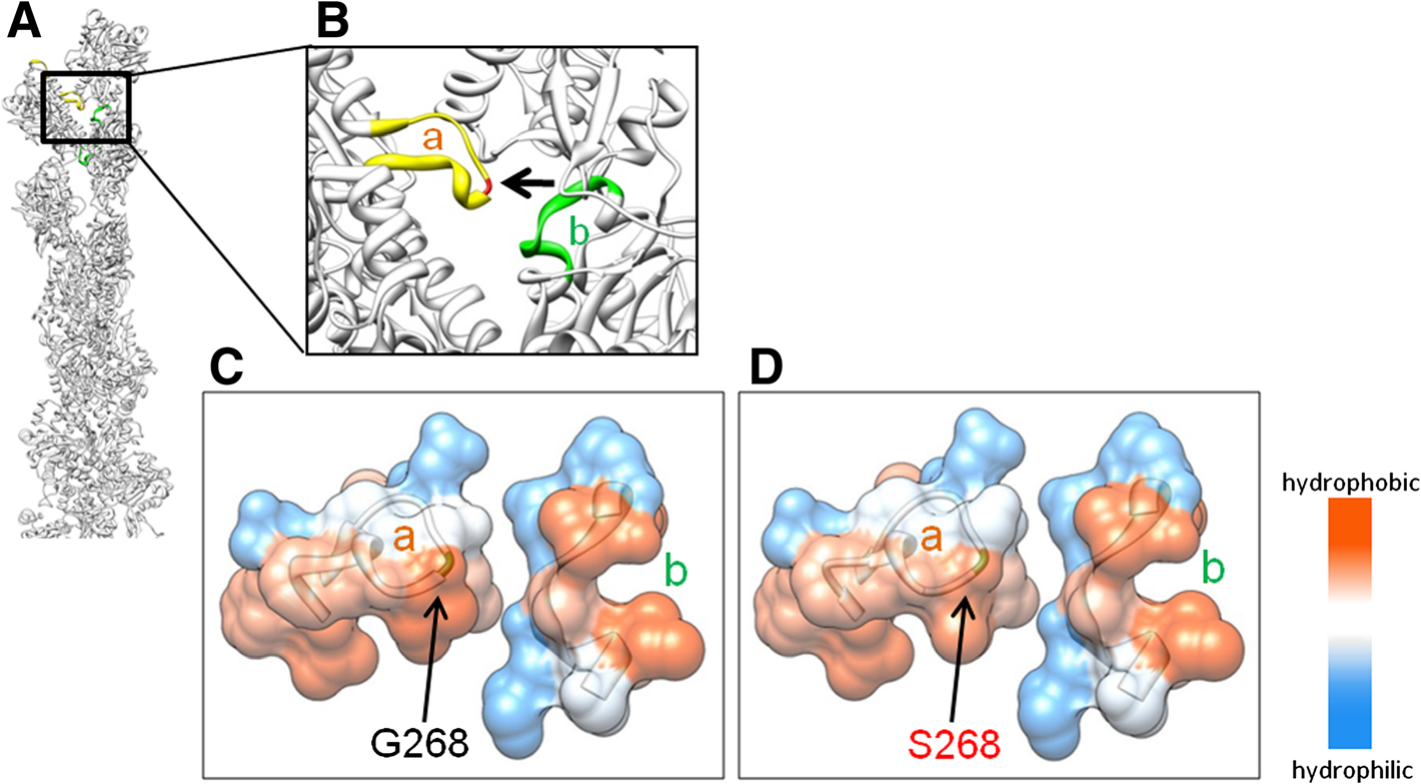
**Molecular modeling of *ACTG *containing the p.G268S mutation. (A)** Ribbon model of filamentous actin gamma 1. **(B)** Magnified ribbon model of filamentous actin gamma 1. Glycine residue 268 is shown in red and indicated by an arrow. Regions in yellow and green indicate the hydrophobic loop (262–274; a) and the corresponding interactive residues (281–289; b), respectively. **(C and D)** Vertical views of the regions a and b superimposed with predicted surface hydrophobicity in the wild type **(C)** and the p.G268S mutant **(D)**.

In family 1 (Figure 1A), subjects III:3 and IV:2 with hearing loss had a unique heterozygous missense mutation of *ACTG1* (c.802G >A; p.G268S), whereas subject III:4 with normal hearing did not. *ACTG1* encodes actin gamma 1 and is responsible for DFNA20/26 (OMIM 604717) [28]. The glycine residue at 268 of actin gamma 1 is located on a hydrophobic loop that has been suggested to be critical for polymerization of the actin monomers into a filament (Figures 2A and 2B) [29]. Molecular modeling predicted that the p.G268S mutation would disrupt the hydrophobic interactions that are important for polymerization of actin gamma 1 (Figures 2C and Figure 2D). The p.G268S mutant would weaken polymerization of actin gamma 1, which could result in destabilized cytoskeletal structure of stereocilia and dysfunction of the sensory hair cells.

Family 2 (Figure 1B) had two candidate genes with possible pathogenic mutations: A unique heterozygous *POU4F3* frameshift mutation, c.1007delC (p.A336Vfs), was detected in subjects III:1 and IV:3 with hearing loss, and a unique heterozygous *DFNA5* nonsense mutation, c.781C >T (p.R261X), was detected in subjects III:2 and IV:3 with hearing loss, whereas subject IV:1 with normal hearing had neither of these mutations. Sanger sequencing revealed that subject IV:2 with hearing loss had both the heterozygous mutations. *POU4F3* is responsible for DFNA15 (OMIM 602459) [30,31], and *DFNA5* is responsible for DFNA5 (OMIM 600994) [32]. A frameshift mutation in *DFNA5*, which would lead to decreased expression, has been reported not to cause hearing loss [33]; therefore, the cause of hearing loss in subjects IV:2 and IV:3 is more likely to *POU4F3* with the p.A336Vfs mutation derived from subject III:1, rather than *DFNA5* with p.R261X mutation derived from subject III:2.

In family 3 (Figure 1C), subjects III:1 and III:2 with hearing loss had compound heterozygous *SLC26A5* with c.209G >A (p.W70X) and c.390A >C (p.R130S) mutations, whereas subjects II:1 and II:2 with normal hearing had a heterozygous p.W70X mutation and a heterozygous p.R130S mutation, respectively. *SLC26A5* encodes prestin, a member of the SLC26A/SulP transporter family, and is responsible for DFNB61 (OMIM 613865) [34].

In family 4 (Figure 1D), subjects I:2 and II:1 with hearing loss did not have candidate mutations in the first 61 genes. Analysis of the additional 23 genes indicated a heterozygous *SIX1* mutation, c.328C >T (p.R110W), in the subjects with hearing loss but not in subject I:1 with normal hearing. *SIX1* is responsible for DFNA23 (OMIM 605192) and Branchio-otic syndrome 3 (BOS3, OMIM 608389). The p.R110W mutation was previously reported in two BOS3 families [35]. To make the clinical diagnosis of branchiootorenal syndrome or branchiootic syndrome, major and minor criteria of these syndromes must be present [36]. In the affected subjects of the present study, clinical histories were thoroughly evaluated and physical examination of the ear, nose, throat, head and neck, and audiological tests were performed. In addition, CT of the temporal bone was evaluated in subject II:1. With these examinations, the affected subjects did not present clinical features of the major and minor criteria other than hearing loss. Therefore, family 4 was considered to have non-syndromic hearing loss, DFNA23, based on the clinical information available at the time of this study.

**Figure 3:**
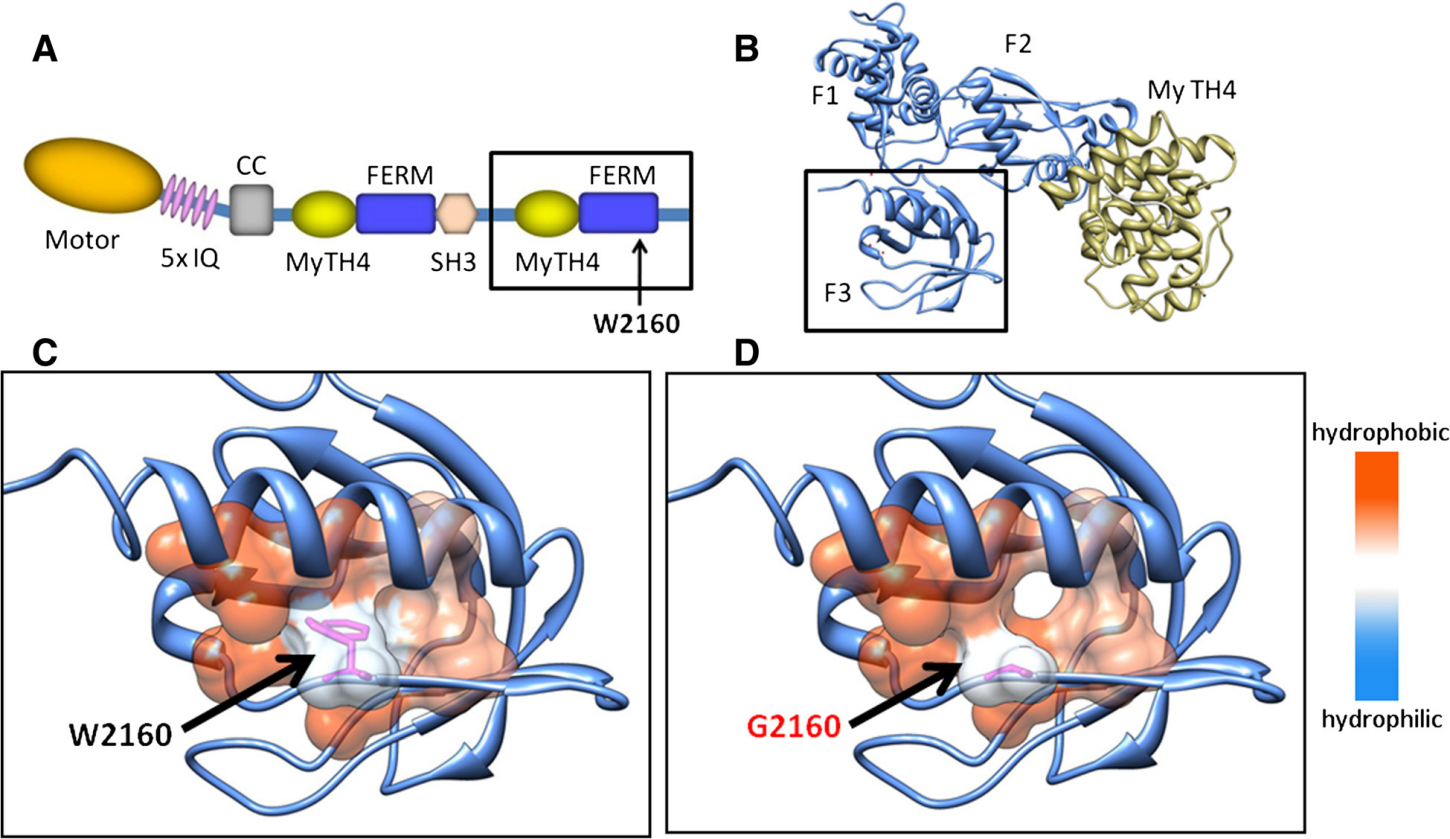
**Molecular modeling of *MYO7A *containing the p.W2160G mutation. (A)** Structural motif of myosin 7A. Tryptophan 2160 on the C-terminal 4.1 protein-ezrin-radixin-moesin (FERM) domain is indicated by an arrow. Motor, myosin motor domain; IQ, Isoleucine-glutamine calmodulin-binding motif; CC, coiled-coil domain; MyTH4, myosin tail homology 4 domain; SH3, Src homology 3 domain. **(B)** Ribbon model of the C-terminal FERM domain consisting of three subdomains (F1, F2, F3) and an MyTH4 domain. **(C, D)** Magnified ribbon model of the F3 subdomain superimposed with predicted surface hydrophobicity in the wild type **(C)** and the p.W2160G mutant **(D)**.

In family 5 (Figure 1E), subjects III:1 and III:2 with hearing loss had compound heterozygous *MYO7A* mutations, c.6439-2A >G (intron 51) and c.6478T >G (p.W2160G). Subjects II:2 and II:4 with normal hearing had a heterozygous c.6439-2A >G mutation and a heterozygous p.W2160G mutation, respectively. *MYO7A* is responsible for DFNA11 (OMIM 601317) [37], DFNB2 (OMIM 600060) [38], and Usher syndrome 1B (OMIM 276900) [39]. Tryptophan 2160 in myosin 7A was found to be located in a carboxyl-terminal FERM domain in the myosin-tail (Figures 3A and Figure 3B); this domain reportedly associates with filamentous actin [40] and contributes to hair bundle formation. Molecular modeling predicted that the p.W2160G mutation would reduce hydrophobic interactions among residues in the center of the F3 subdomain of the FERM domain (Figures 3C and 3D). The p.W2160G mutation would destabilize the structure of the F3 domain and could result in disrupted protein interaction and stereocilial degeneration of the sensory hair cells [41,42].

In family 6 (Figure 1F), subjects II:1 and II:2 with hearing loss had a heterozygous *CDH23* mutation, c.719C>T (p.P240L), and a heterozygous *PCDH15* mutation, c.848G >A (p.R283H). Sanger sequencing revealed that the other subject with hearing loss (subject II:3) also had both heterozygous *CDH23* and *PCDH15* mutations. A p.P240L mutation in*CDH23* has been reported to be pathogenic [43]. Subject I:1 with normal hearing had a heterozygous mutation in *CDH23* (p.P240L), and subject I:2 with normal hearing had a heterozygous mutation in *PCDH15* (p.R283H). *CDH23* is responsible for both DFNB12 (OMIM 601386) and Usher syndrome 1D (OMIM 601067) [44], whereas *PCDH15* is responsible for both DFNB23 (OMIM 609533) and Usher syndrome 1F (OMIM 602083) [45]. Double heterozygous mutations of *CDH23* and *PCDH15* have been reported to be a digenic cause of hearing loss [46].

In family 7 (Figure 1G), subjects II:1 and II:2 with hearing loss did not have candidate mutations in the first 61 genes. Analysis of the additional 23 genes indicated a compound heterozygous *USH2A* variant or mutation, c.1346G >A(p.R449H) and c.12431delC (p.A4144GfsX23), in subjects with hearing loss, whereas subjects I:1 and II:2 with normal hearing had a heterozygous p.R449H variant and a heterozygous p.A4144GfsX23 mutation, respectively. *USH2A* is responsible for Usher syndrome 2A (OMIM 276901) [47]. Although *USH2A* with the p.R449H variant was not found on dbSNP135, 1000GENOME, or the Exome Variant Server, the allele frequency in Japanese control subjects with normal hearing was 1.3% (5/378).

In the remaining eight families, none of the detected variants co-segregated with hearing loss in the pedigrees (data not shown).

## Discussion

In the present study we selected Japanese subjects that had hereditary hearing loss without *GJB2* mutations, mitochondrial mutations, enlarged vestibular aqueduct or auditory neuropathy-associated *OTOF* mutations, and we aimed to detect the spectrum of rare deafness genes in these patients. Targeted NGS for 84 deafness genes resulted in identification of candidate genes in 7 of 15 families and revealed the diverse spectrum of rare deafness genes in Japanese subjects with nonsyndromic hearing loss for the first time. This is the first report of mutations in *ACTG1*, *POU4F3*, and *SLC26A5* in Japanese families with hearing loss. Families 5, 6, and 7 appeared to have candidate mutations or variants in *MYO7A*, *CDH23*, *PCDH15*, and *USH2A*, all of which are associated with Usher syndrome [39,44,45,47]. Our results are in contrast to an NGS study of a different ethnic group [48], which showed *TMC1* mutations to be the prevalent candidate cause of hearing loss.

For the eight families without candidate genes, hearing loss could be attributable to mutations in non-captured regions including regulatory domains of the 84 genes, other unidentified deafness genes, unknown multigenic causes, copy number variations, or chromosomal structural change.

### Double heterozygous mutations

In family 5, double heterozygous mutations of *CDH23* and *PCDH15* were detected as a candidate cause. This combination of double heterozygous mutations has been reported [46]. Cadherin 23 and protocadherin 15 consist of the upper and lower part of tip link, respectively, which is critical for proper function of mechanotransduction channels on the stereocilia of the sensory hair cells [49]. In addition, P240 of CDH23 is on the extracellular cadherin 1 domain, and R283 of PCDH15 is on the extracellular cadherin 2 domain, which are considered to interact with each other for tip-link bound [49], raising the possibility that the double heterozygous mutations could lead to a destabilized tip-link.

Additional findings of double heterozygous mutations associated with hereditary hearing loss have been reported for *KCNJ10* and *SLC26A4*[50] and for *FOXI1* and *SLC26A4*[51], and some mutated genes may have a modifying effect [52]. Although most NGS pipelines, including ours, focus on identifying monogenic causes of disease, development of a detection strategy for digenic and oligogenic causes of disease should be considered in the future.

### Discrimination of mutations from variants

The key challenge for the diagnostic application of NGS is to distinguish causal alleles from the numerous nonpathogenic variants present in each individual. In the present study, for example, the high allele frequency of *USH2A* with the p.R449H variant in Japanese control subjects implied that pathogenicity of this variant was unlikely. Ethnic diversity of genetic variance has been reported in deafness genes such as *OTOF*[12] and *CDH23*[43,53], and integration of a database of genetic variants with allele frequencies in a specific ethnic group would increase the certainty of the causative nature of genetic mutations by filtering out variants that occur with high frequency. This would facilitate targeted NGS analysis for genetic diagnosis of hearing loss.

## Competing interests

The authors declare that they have no competing interests.

## Authors’ contributions

HM and NS carried out capturing and sequencing the DNA samples, interpreted the data, and drafted the manuscript. CT carried out capturing and sequencing the DNA samples. AS and JK worked on DNA sequencing and interpreting the data. KN carried out molecular modeling of gene products. KKosaki and TM designed the study and interpreted the data. NM, KKaga, and TM contributed to accumulation and interpretation of clinical data. TM finalized the manuscript. All authors read and approved the final manuscript.

## Supplementary Material

Additional file 1 The 84 genes that were targeted for next-generation sequencing. Click here for file

Additional file 2 Clinical features of family members. Click here for file

Additional file 3 **Audiograms of subjects with hearing loss in the seven families in which candidate genes were detected.** Figure legend: Hearing level as a function of frequency in subject IV:2 from family 1 (A), subject III:3 from family 1 (B), subject IV:3 from family 2 (C), subject III:1 from family 2 (D), subject III:2 from family 2 (E), subject III:1 from family 3 (F), subject II:1 from family 4 (G), subject III:1 from family 5 (H), subject II:2 from family 6 (I), subject II:3 from family 6 (J), and subject II:2 from family 7 (K). Open circles with solid lines represent air conduction thresholds of the right ear; crosses with dotted lines represent air conduction thresholds of the left ear; [ symbols represent bone conduction thresholds of the right ear; ] symbols represent bone conduction thresholds of the left ear; arrows pointing to the bottom left represent scale-out hearing level of the right ear; arrows pointing to the bottom right represent scale-out hearing level of the left ear. Click here for file
