# Supplementary material for: Diverse spectrum of rare deafness genes underlies early-childhood hearing loss in Japanese patients: a cross-sectional, multi-center next-generation sequencing study
Source: Orphanet J Rare Dis. 2013 Oct 28;8:172. doi: 10.1186/1750-1172-8-172 (PMC4231469; doi:10.1186/1750-1172-8-172)
Supplement: Additional file 2 — Clinical features of family members. [file 1750-1172-8-172-S2.doc]

**Additional file 2.** **Clinical features of family members.**

Family 1 exhibited an autosomal dominant inheritance pattern of nonsyndromic hearing loss (Figure 1A). The proband (subject IV:2) was diagnosed with mild hearing loss at age 10 years [Additional file 3A]. At the time of the study, subject IV:2 was 16 years old and had shown no progression of hearing loss. The father (subject III:3) was recognized as having hearing difficulties at age 45 years. At the time of the study, the father was 53 years old and had progressive hearing loss with sloping at high frequency [Additional file 3B]. Other subjects in the family showed progressive hearing loss that started between the ages of 20 and 40 years.

The inheritance pattern of hearing loss in family 2 could be interpreted as autosomal dominant (Figure 1B). The proband (subject IV:3) passed newborn screening but showed severe progressive sensorineural hearing loss at age 1 year, 11 months [Additional file 3C]. His sibling (subject IV:2) showed congenital severe progressive sensorineural hearing loss. Although subject III:1 showed profound hearing loss [Additional file 3D], subject III:2 exhibited moderate mixed conductive-sensorineural hearing loss [Additional file 3E].

Family 3 exhibited an autosomal recessive inheritance pattern of hearing loss (Figure 1C), with the proband (subject III:1) showing severe hearing loss [Additional file 3F] and subject III:2 showing moderate hearing loss. Subject II:2 originated from Indonesia. Subject III:2 was referred to the study based on newborn hearing screening.

Family 4 exhibited an autosomal dominant or mitochondrial inheritance pattern of hearing loss (Figure 1D). Both the proband (subject II:1) [Additional file 3G] and mother (subject I:2) showed congenital profound hearing loss but they did not show other clinical features of major and minor criteria which must be present to make the clinical diagnosis of branchiootorenal (BOR) syndrome and branchiootic syndrome (BOS ) [36].

Family 5 exhibited an autosomal recessive inheritance pattern of hearing loss (Figure 1E). The proband (subject III:1) [Additional file 3H] and the dizygotic twin (subject III:2) showed congenital severe hearing loss. Both subjects showed delayed onset of walking and other motor activities, probably owing to a vestibular disorder.

Family 6 exhibited an autosomal recessive inheritance pattern of progressive hearing loss (Figure 1F). The proband (subject II:2) [Additional file 3I] and her sibling (subject II:1) showed progressive, severe-to-profound mixed conductive-sensorineural hearing loss. The proband also exhibited vertigo. Subject II:3, the monozygotic twin of the proband, showed progressive, severe mixed conductive-sensorineural hearing loss in her left ear and mild hearing loss that was steeply sloping at high frequencies in her right ear [Additional file 3J]. The onset of hearing loss was at age 5 years for subject II:1 and congenital for subjects II:2 and II:3.

Family 7 exhibited an autosomal recessive inheritance pattern of hearing loss (Figure 1G). The proband (subject II:2) showed congenital severe hearing loss [Additional file 3K], whereas her sibling (subject II:1) showed moderate hearing loss. Neither subject exhibited vestibular dysfunction.
