# Supplementary figures and images for: Diverse spectrum of rare deafness genes underlies early-childhood hearing loss in Japanese patients: a cross-sectional, multi-center next-generation sequencing study
Source: Orphanet J Rare Dis. 2013 Oct 28;8:172. doi: 10.1186/1750-1172-8-172 (PMC4231469; doi:10.1186/1750-1172-8-172)

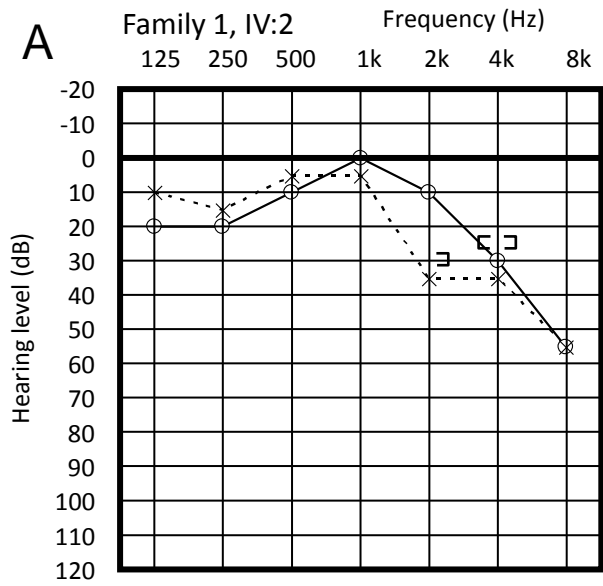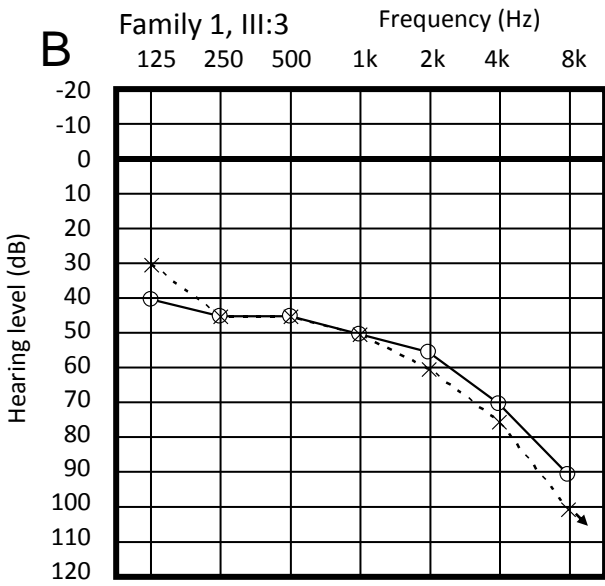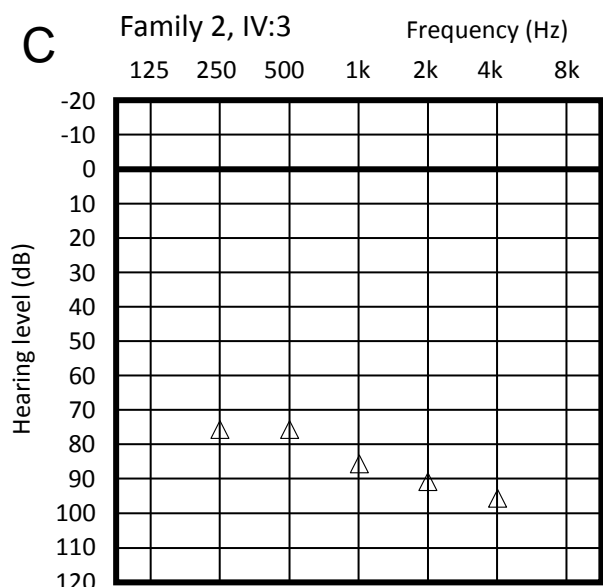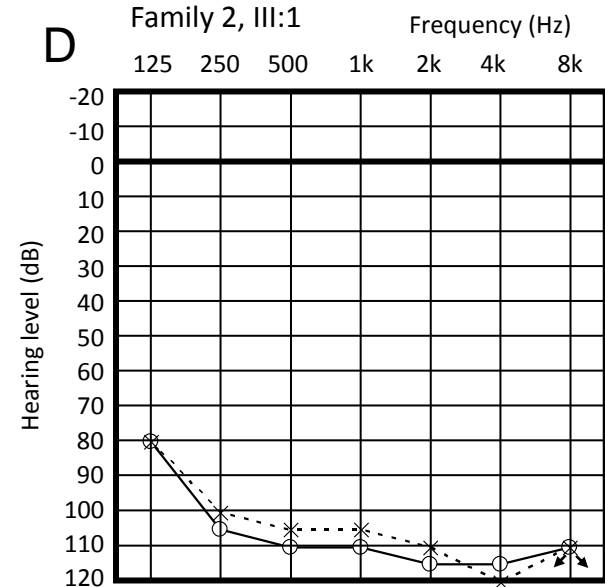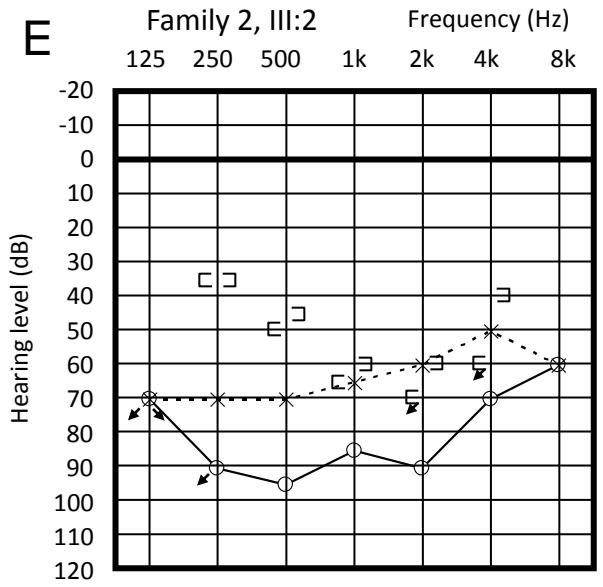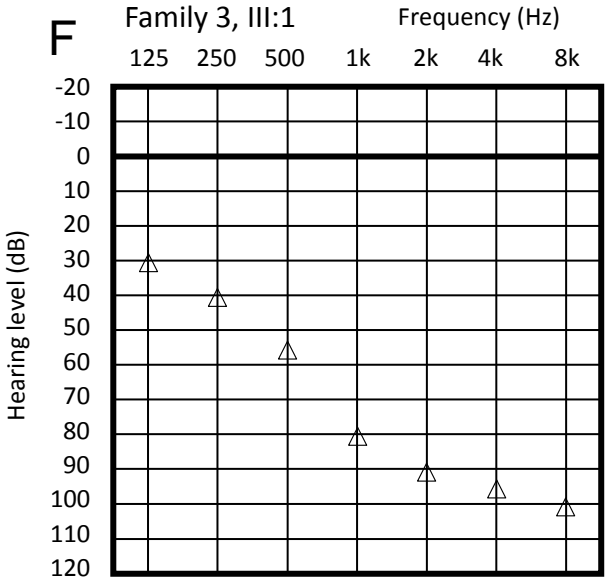

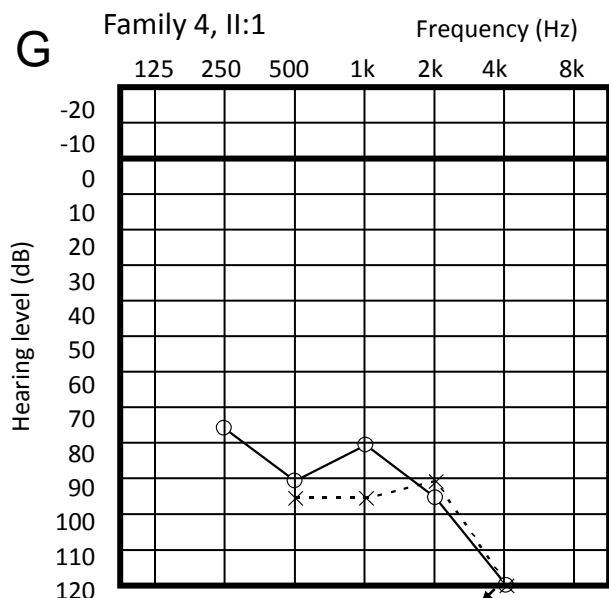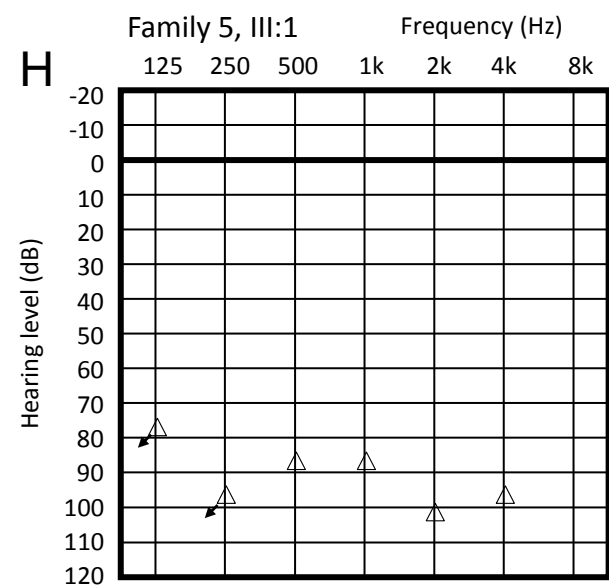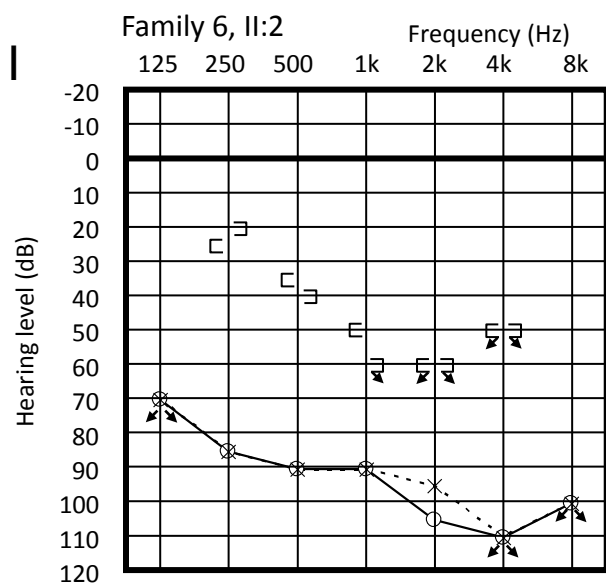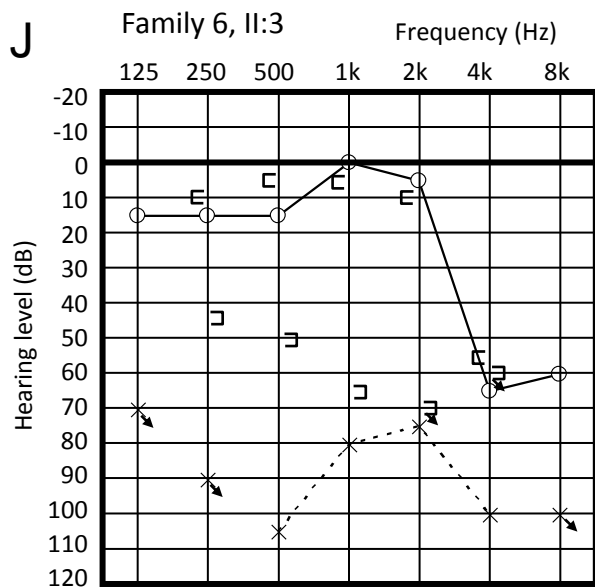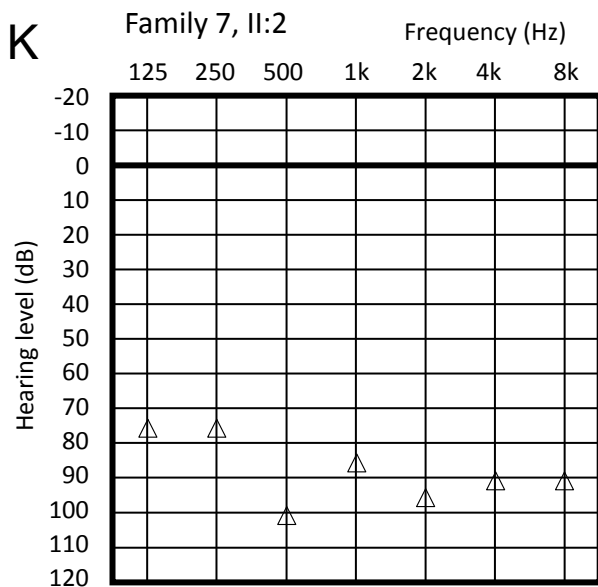

Supplement: Additional file 3 — Audiograms of subjects with hearing loss in the seven families in which candidate genes were detected. Figure legend: Hearing level as a function of frequency in subject IV:2 from family 1 (A), subject III:3 from family 1 (B), subject IV:3 from family 2 (C), subject III:1 from family 2 (D), subject III:2 from family 2 (E), subject III:1 from family 3 (F), subject II:1 from family 4 (G), subject III:1 from family 5 (H), subject II:2 from family 6 (I), subject II:3 from family 6 (J), and subject II:2 from family 7 (K). Open circles with solid lines represent air conduction thresholds of the right ear; crosses with dotted lines represent air conduction thresholds of the left ear; [ symbols represent bone conduction thresholds of the right ear; ] symbols represent bone conduction thresholds of the left ear; arrows pointing to the bottom left represent scale-out hearing level of the right ear; arrows pointing to the bottom right represent scale-out hearing level of the left ear. [file 1750-1172-8-172-S3.pdf]
